# *In vitro* and *in vivo* anti-lymphoma effects of *Ophiorrhiza pumila* extract

**DOI:** 10.18632/aging.204041

**Published:** 2022-05-03

**Authors:** Lixia Fan, Wanqin Liao, Zezhen Chen, Shaojing Li, Anping Yang, Min-Min Chen, Hui Liu, Fang Liu

**Affiliations:** 1 Department of Basic Medicine and Biomedical Engineering, School of Medicine, Foshan University, Foshan, Guangdong, China

**Keywords:** *Ophiorrhiza pumila* extract, lymphoma, anti-cancer activity, proliferation, EGFR

## Abstract

Background: Current therapeutic strategies on patients with lymphomas remains limited. Previously we found the suppressive effect of *Ophiorrhiza pumila* (OPE) on hepatocarcinoma. In present study, the effect of OPE on lymphoma *in vitro* and *in vivo* were investigated.

Methods: CCK-8 assay was applied to detect the effect of OPE on cell proliferation. Flow cytometry was used to analyze the effect of OPE on cell cycle distribution and apoptosis. Xenograft mouse model was conducted to determine the anti-tumor activity of OPE. TNUEL assay was performed to detect the apoptosis in tumor tissues. Western blot and immuno-histochemistry were used to determine protein expression.

Results: *In vitro* tests indicate that OPE suppressed A20 cell proliferation in a dose- and time-dependent manner. OPE treatment induced cell cycle arrest at S phase and elevated apoptosis in A20 cells. OPE displayed a significant inhibition in tumor growth in a mouse xenograft model. OPE promoted apoptosis of tumor cell in the mouse model Cleaved caspase 3 expression and Bax/Bcl2 ratio were also enhanced. In addition, OPE suppressed A20 cell viability partially by reducing phosphorylation of EGFR.

Conclusions: Our data showed that OPE suppressed the proliferation of lymphoma cells and promoted apoptosis *in vitro* and *in vivo*, which might be partially mediated by inactivating EGFR signaling.

## INTRODUCTION

Lymphomas are a heterogeneous group of molecularly, biologically and clinically distinct lymphoproliferative malignancies [1]. Multiple therapies, such as chemotherapy, radiotherapy, immunotherapy and target therapy, have been developed for the treatment of lymphomas [2]. Although promising effects have been achieved by approaches, relapse and drug resistance are common. Therefore, developing novel strategies for lymphomas remains a primary concern currently [3, 4]. We had previously identified that the soluble form of CXCL16 and TNF-α may be used as prognostic markers and their combination could be a promising approach in the context of diffuse large B-cell lymphoma therapy [3, 4].

Anti-cancer agents derived from natural plants have been reported to exhibit low toxicity and effective therapeutic activity in different types of tumors [5–7]. *Ophiorrhiza pumila* (*O. pumila*) is a Rubiaceae family plant that grows in many Asia countries, such as Japan, Vietnam, Philippines and China [8]. *O. pumila* has been considered to be a valuable alternative source of camptothecin (CPT), which is widely used to treat various cancers, such as colorectal, ovarian and lung cancer [9, 10]. Studies refer to the biosynthesis process of CPT in *O. pumila* are decumulating [8, 11, 12], but the function of *O. pumila* compounds in cancer have rarely been explored. Previously, we reported that treatment with *O. pumila* extract (OPE) suppresses the proliferation and migration of liver cancer cells, indicating an anti-cancer activity of OPE in hepatocarcinoma [13]. However, the effect of compounds of *O. pumila* on other types of cancers remains unknown.

In this study, we aimed to investigate the cytotoxicity of OPE in lymphomas *in vitro* and *in vivo*, which may expand our understanding of the anti-cancer activity of OPE and may provide data for the discovery of novel compounds against B cell lymphomas from *O. pumila*.

## RESULTS

### OPE suppresses the proliferation and induces S phase arrest in A20 cells

**Figure 1:**
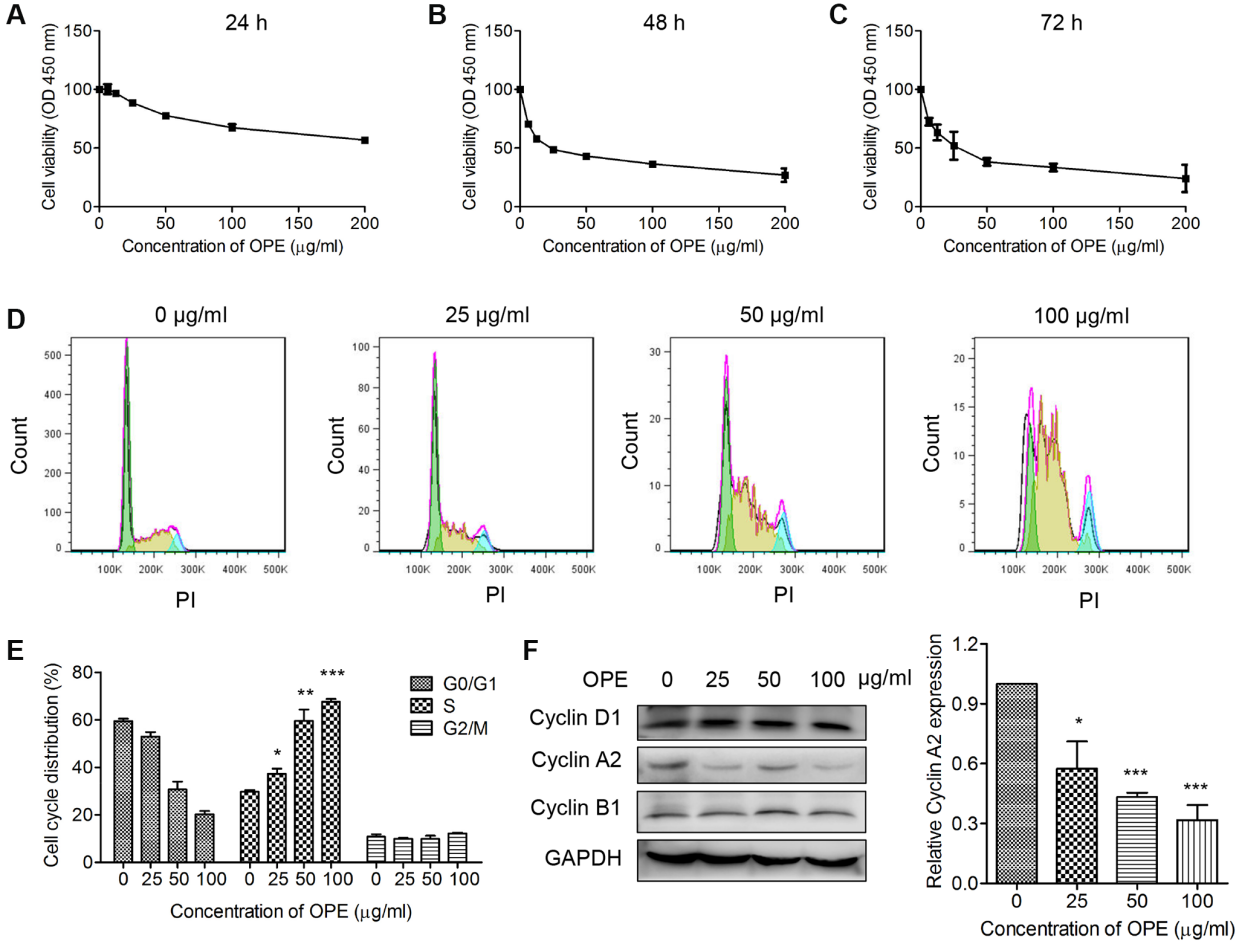
**OPE inhibits the proliferation and induces S phase arrest in A20 cells.** (**A**–**C**) OPE inhibits the proliferation of A20 cells. A20 cells were treated with different concentrations of OPE (0, 6.25, 12.5, 25, 50, and 100 μg/mL) for 24 h (**A**), 48 h (**B**), and 72 h (**C**), respectively, and the cell viability was examined by CCK-8 assay. (**D**, **E**) OPE induces S-phase arrest in A20 cells. A20 cells were treated with different concentrations of OPE (0, 25, 50, and 100 μg/mL) for 48 h, and cell cycle distribution was accessed by flow cytometry. (**F**) Western blot was carried out to detect the expression of cell cycle-associated proteins. Data are presented as means ± SD of at least three independent experiments. (^*^p < 0.05; ^**^p < 0.01; ^***^p < 0.001, compared to the untreated control).

To determine the effect of OPE on cell viability, CCk-8 assay was performed in A20 cells treated with different concentrations of OPE for 24 h, 48 h, and 72 h, respectively. The results showed that treatment with OPE significantly reduced A20 cell viability, which was in a time- and dose-dependent manner (Figure 1A–1C). The IC_50_ value (50% inhibition) of OPE was 223.75 μg/mL at 24 h, 27.95 μg/mL at 48 h, and 26.4 μg/mL at 72 h.

Cell cycle arrest is an important event related to cell growth. Hence, OPE may affect the viability of A20 cells by inducing cell cycle arrest. Flow cytometry analysis showed that administration of OPE significantly altered the cell cycle distribution. The percentages of A20 cells at S phase for 0 μg/mL, 25 μg/mL, 50 μg/mL, and 100 μg/mL groups were 29.7%, 37.3%, 59.5%, and 67.5%, respectively (Figure 1D and 1E). In consistent with these results, Western blot analysis showed that the expression of Cyclin A2, a key mediator of S phase, was markedly reduced (Figure 1F). Together, these results suggest that OPE could induce S phase cell cycle arrest in A20 cells.

### OPE triggers apoptosis of A20 cells

**Figure 2:**
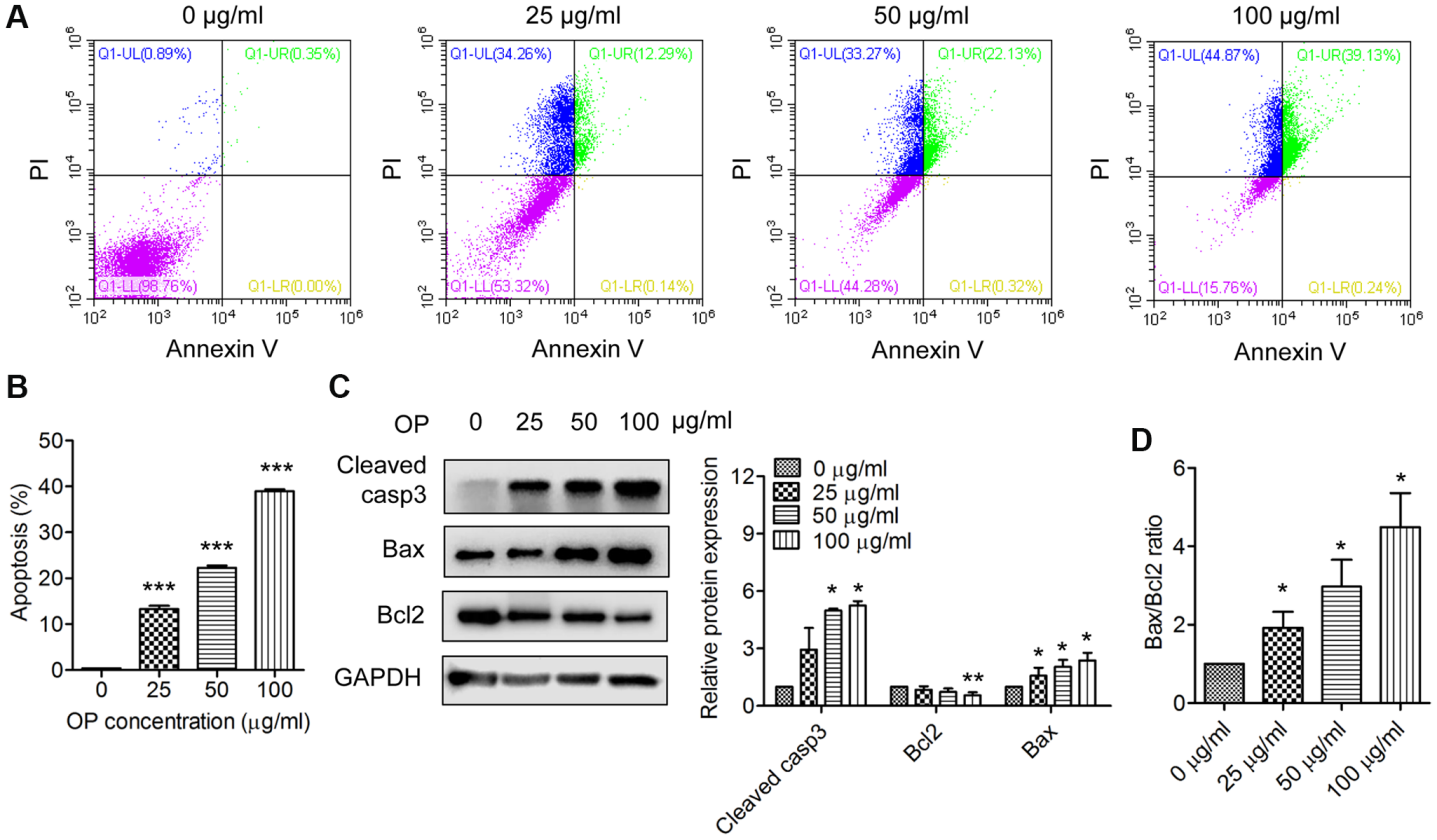
**OPE enhances apoptosis in A20 cells.** (**A**, **B**) Flow cytometry analysis of apoptosis of A20 cells after treatment with OPE (0, 25, 50, 100 μg/mL) for 48 h. (**C**) Western blot analysis of the expression levels of apoptosis-related proteins. A20 cells were treated with OPE (0, 25, 50, and 100 μg/mL) for 48 h, and Western blot was conducted with the indicated antibodies. (**D**) The alteration of the Bax/Bcl2 ratio in A20 cells following treatment with OPE. Data are presented as means ± SD of at least three independent experiments. (^*^p < 0.05; ^***^p < 0.001, compared to the untreated control).

Apoptosis is a key process in regulating cell death. Therefore, we wondered whether OPE had an effect on A20 cell apoptosis. A20 cells were treated with different concentrations of OPE, and the apoptotic rate was ascertained by flow cytometry. Exposure to OPE led to a remarkable increase in apoptotic cell population, which was in a dose-dependent manner (Figure 2A and 2B). The percentages of apoptotic cells in 0 μg/mL, 25 μg/mL, 50 μg/mL, and 100 μg/mL groups were 0.38%, 13.27%, 22.28%, and 38.95%, respectively. In consistent with these results, OPE treatment significantly elevated the expressions of apoptosis proteins, Bax and cleaved-caspase 3, but had no significant effect on Bcl2 expression (Figure 2C). The expression ratio of Bax/Bcl2 was increased after OPE exposure (Figure 2D). Together, these results indicate that OPE suppressed A20 cell growth via triggering apoptosis.

### OPE represses A20 cell growth *in vivo*

**Figure 3:**
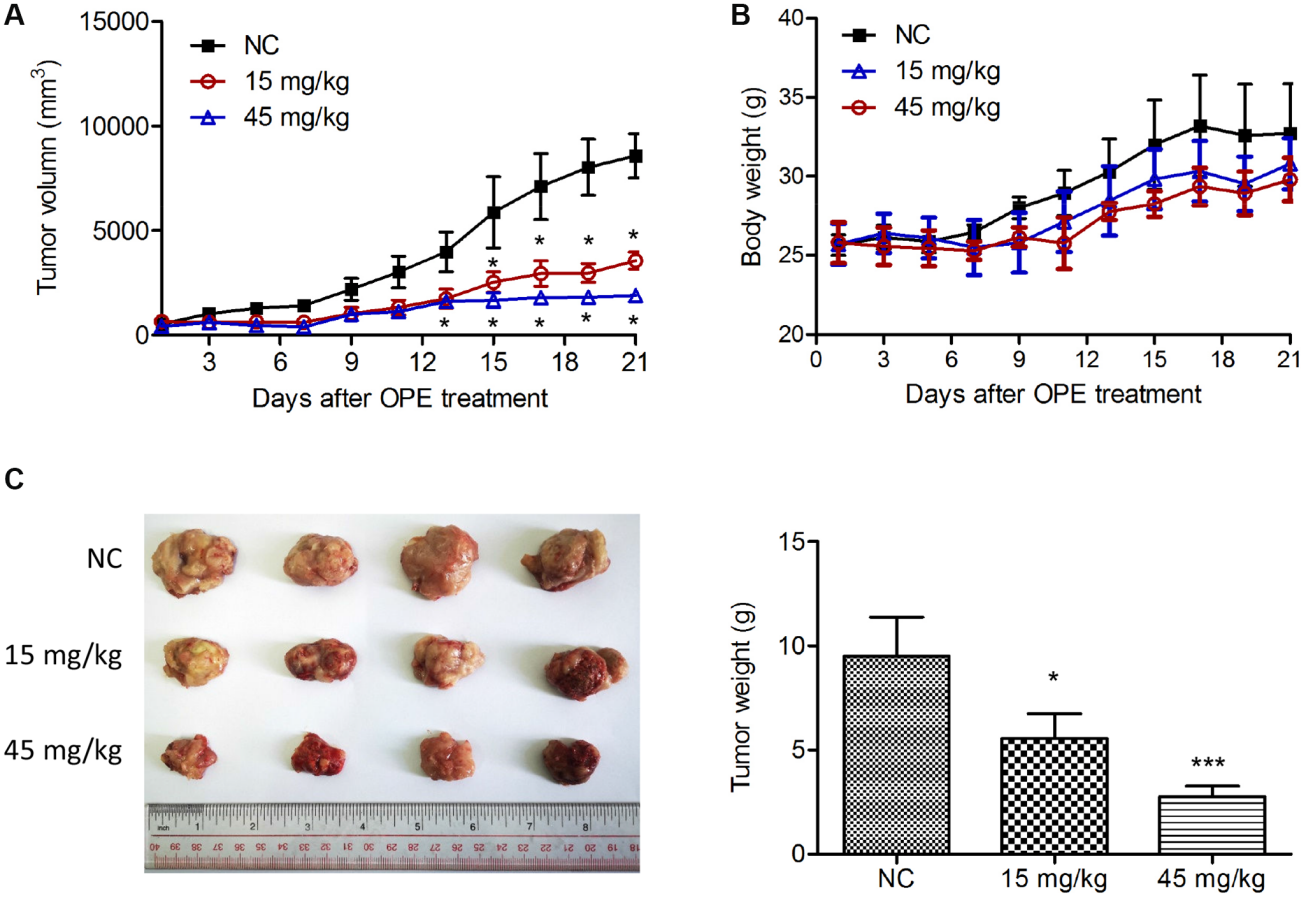
**OPE suppresses A20 cell growth *in vivo*.** (**A**) The effect of OPE on the volume of tumor derived by A20 cells. A20 cells were subcutaneously injected into the right oxter of Balb/c mice followed by the treatment with DMSO (NC), 15 mg/kg, or 45 mg/kg OPE (*n* = 4). The tumor volume was measured every other day. (**B**) The body weight of Balb/c mice after tumor cell inoculation and treatment. (**C**) The representative images of isolated tumors from Balb/c mice. Data are presented as means ± SD. (^*^p < 0.05; ^***^p < 0.001, compared to the untreated control).

Next, we determine whether OPE had an anti-lymphoma activity *in vivo* by using a xenograft mouse model. Treatment with OPE significantly decreased the tumor development of derivate from A20 cells, which was also in a time- and dose-dependent manner (Figure 3A and 3C). The inhibitory rates at Day 21 for 15 mg/kg and 45 mg/kg were 58.4% and 77.9%, respectively (Figure 3A). There was no significant difference in the body weight among different groups (Figure 3B).

### OPE induces apoptosis in A20-derived tumors

**Figure 4:**
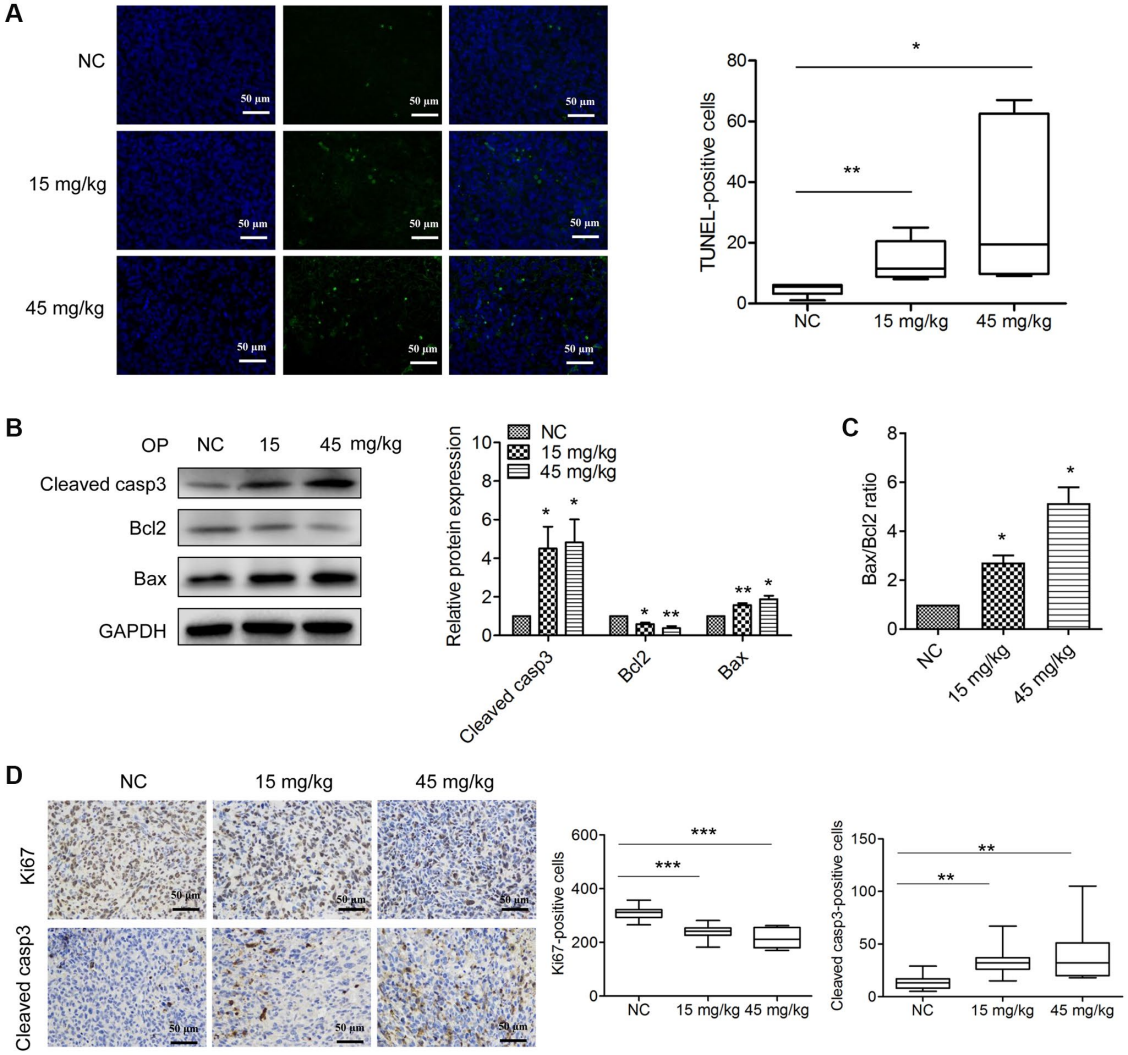
**OPE induces apoptosis in A20-derived tumors.** (**A**) The effect of OPE on tumor apoptosis. The tumor apoptosis was evaluated by TUNEL staining (green). DAPI (blue) was used to stained nuclei. (**B**) Western blot analysis of apoptosis-related proteins in tumor tissues. (**C**) The alteration of the Bax/Bcl2 ratio in tumor tissues following treatment with OPE. (**D**) Immunohistochemistry staining analysis of the cleaved caspase3-positive cells and Ki67-postive cells in tumor tissues. Data are presented as means ± SD of at least three independent experiments. (^*^p < 0.05; ^**^p < 0.01; ^***^p < 0.001, compared to the untreated control).

To access the effect of OPE on the apoptosis in A20-derived xenografts, TUNEL staining was performed. Consistent with the *in vitro* results, increased TUNEL-positive cells were observed in OPE-treated groups (15 mg/kg and 45 mg/kg) compared with the NC group (Figure 4A). Western blot analysis of the tumor tissue samples also showed that the expression levels of cleaved caspase 3 and Bax were dose-dependently increased following the treatment of OPE, while no significance in the expression of Bcl2 was observed (Figure 4B). Consistently, treatment with OPE resulted in an increase in the expression ratio of Bax/Bcl2 in tumor tissues (Figure 4C). In agreement with these results, immuno-histochemistry staining showed that there were more cleaved caspase3-positive cells and fewer Ki67-postive cells in OPE-treated groups than in the NC group (Figure 4D). Together, these results suggest that OPE induces A20 cell apoptosis *in vivo*.

### OPE suppresses A20 cell proliferation via inactivation of EGFR

**Figure 5:**
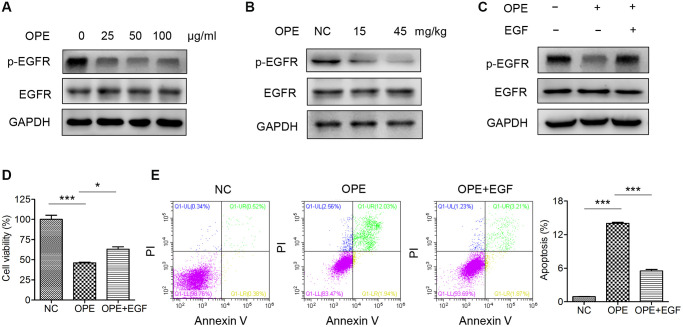
**OPE suppresses A20 cell proliferation via inactivation of EGFR.** (**A**) Western blot analysis of the expression and phosphorylation of EGFR in A20 cells. (**B**) Western blot analysis of the expression and phosphorylation of EGFR in A20 cell-derived tumors. (**C**) The viability of A20 cells after treatment with OPE (50 μg/mL) together with or without EGF (50 ng/mL). (**D**) The apoptosis of A20 cells after treatment with OPE (50 μg/mL) together with or without EGF (50 ng/mL). (**E**) Flow cytometry analysis of apoptosis of A20 cells treatment with OPE (μg/mL) together with or without EGF (50 ng/mL) for 48 h. Data are presented as means ± SD of at least three independent experiments. (^*^p < 0.05; ^***^p < 0.001, compared to the untreated control).

EGFR signaling plays a vital role in the regulation of apoptosis. Therefore, we investigated whether OPE had an effect on the activation of EGFR. Indeed, Western blot analysis showed that administration of OPE remarkable reduced the phosphorylation of EGFR in A20 cells (Figure 5A). Also, the phosphorylation of AKT, a downstream target of EGFR signaling, was reduced after OPE treatment (Supplementary Figure 1). Consistently, the level of p-EGFR in tumors isolated from OPE-treated mice (15 mg/kg and 45 mg/kg) was significantly decreased (Figure 5B). Treatment with EGF could partially restore the cell viability of A20 cells (Figure 5C and 5D). Moreover, administration of EGF restrained the enhanced effect of OPE on the apoptosis rate of A20 cells (Figure 5E). Together, these results implied that EGFR suppression partially accounted for the anti-proliferative activity of OPE in A20 cells.

## DISCUSSION

Exploring the functional activity of the extract of a certain plant is an important step for discovering novel anti-cancer agents. In addition to alkaloids, *O. pumila* also produce anthraquinones, glucosides, and chlorogenic acid, which are potential chemoprotective compounds against cancers [10, 14, 15]. In our research, the dried whole plant of *O. pumila* were crushed, and extracted by 95% (v/v) ethanol, the extract solution was concentrated and produced ethanol extract (OPE). Although we have reported that OPE has an anti-liver cancer activity [13], its activity in lymphomas remains unclear. In present study, we found that OPE inhibits the proliferation and induces cell cycle arrest and apoptosis in A20 cells. Moreover, OPE suppresses A20 tumor growth *in vivo*. Thus, our findings highlight an anti-lymphoma activity of OPE.

Anti-cancer compounds commonly trigger tumor cell death via inducing cell cycle arrest [16–18]. For example, the ethanolic extract of *Cordyceps cicadae* exerts its antitumor activity in gastric cancer cells by inducing S phase arrest [19]. Withaferin A suppresses glioblastoma cell growth in triggering G2/M arrest [20]. In our study, OPE induced a S phase arrest in A20 cells. Of note, previously we reported that OPE could induced a G2/M arrest in liver cancer cells [13]. Thus, these results indicate that the action of OPE on cell cycle distribution is cell type-dependent.

Given the critical role of apoptosis in cancer cell survival [21], we also accessed the effect of OPE on apoptosis. As expected, a significantly increased number of apoptotic cells was visualized in OPE-treated group compared with the control group. Consistent with the *in vitro* result, TUNEL assay also showed a higher apoptotic rate in A20 tumor tissues isolated form OPE-treated mice compared with those from the control mice. Furthermore, Western blot analysis showed that the expression of apoptosis-related proteins, cleaved caspase 3 and Bax, two key mediators in the apoptosis process [22, 23], were significantly elevated, confirming the enhanced effect of OPE on A20 cell apoptosis.

A series of signaling pathways had been report involved in the proliferation, apoptosis, and survival of lymphoma cells, including EGFR signaling [24–27].

EGFR is a member of ErbB family which plays vital roles in many processes associated with tumor development, such as proliferation, survival, migration and apoptosis [28, 29]. The PI3K/AKT signaling pathway is considered as an attractive target for the development of new anticancer agents [30]. EGFR is also an upstream protein in the PI3K/AKT signal transduction pathway, which is an important target in cancer research [31]. Thus, targeting EGFR/AKT signaling is considered to be a crucial strategy of cancer therapy [32]. Recent evidence has revealed that EGFR signaling is implicated in the progression of lymphoma. It has been reported that EGFR activation contributed to PDGFD induced-ibrutinib resistance in diffuse large B-cell lymphoma (DLBCL) [33]. LncRNA TUC338 promotes the proliferation of DLBCL cells via activating EGFR pathway [34]. These studies indicate that the activation of EGFR signaling confers the malignancy of DLBCL. Our data showed that OPE could significantly reduce the phosphorylation of EGFR, as well as the phosphorylation of AKT. The suppression of EGFR signaling could induce apoptosis and lead to cell death, consistent with previous studies [35, 36]. Moreover, restoration of EGFR activity partially reversed the effects of OPE on cell viability and apoptosis. Hence, our results indicate that EGFR suppression contributes to the anti-proliferative effect of OPE in A20 cells.

## CONCLUSIONS

In conclusion, OPE mediated A20 cell growth suppression by inducing apoptosis and cell cycle arrest. In addition, OPE displayed a significant inhibition in tumor growth in a mouse model, which might be related to enhanced cleaved caspase 3 expression and Bax/ Bcl2 ratio. Moreover, OPE exerts its proliferation-suppressive activity in A20 cells involves in inactivation of EGFR. Our findings imply that OPE might be a promising target for lymphoma therapy. However, the molecular mechanisms of the anti-lymphoma activity of OPE still needs to be further investigated.

## MATERIALS AND METHODS

### Reagents and materials

Antibodies against cleaved caspase-3, Bcl-2, Bax, GAPDH, and HRP-conjugated secondary antibodies were purchased from Cell Signaling Technology (USA). Antibodies against Cyclin D1, Cyclin A2, Cyclin B1 were purchased from Proteintech (USA). OPE was obtained as reported previously (Supplementary Figure 2 and Supplementary Table 1) [13]. OPE was dissolved in methanol to make the 100 mg/mL solution and stored at −20°C until use.

### Cell culture

A murine lymphoma cell line, A20 cell line, which was pathologically mimic the diffuse large B cell lymphoma, were purchased from the American Type Culture Collection (ATCC, USA). A20 cells were maintained in 1640 medium plus 10% FBS and 1% penicillin/streptomycin and were incubated in an incubator with 5% CO_2_ at 37°C.

### Cell viability analysis

CCK-8 assay was performed to determine the viability of A20 cells after treatment with OPE. In brief, A20 cells (8~1.2 × 10^3^ per well) in 100 μL completed 1640 medium were placed in 96-well plates and were exposed to different concentrations of OPE (0, 6.25, 12.5, 25, 50, 100, and 200 μg/ml). After treatment for 24 h, 48 h, and 72 h, 10 μL of CCK-8 reagent (Dojindo, Japan) was added to each well and incubation for 2~4 h. The absorbance was then measured at 450 nm on a microplate spectrophotometer.

### Cell cycle analysis

Flow cytometry was applied to investigate the influence of OPE on cell cycle distribution as described previously [13]. Briefly, A20 cells were incubated with different concentrations of OPE (0, 25, 50, and 100 μg/mL). After treatment for 48 h, A20 cells were harvested, washed with PBS, and stained using the Cell Cycle Staining Kit (MultiSciences, China). The distribution of cell cycle was analyzed by the CytoFlex-LX flow Cytometer (Beckman, USA).

### Apoptosis analysis

The apoptosis of A20 cells treated with OPE was detected by using the Annexin V-FITC apoptosis detection kit (BD, USA). A20 cells (3 × 10^5^ cells per well) were placed in 6-well plates and were exposed to different concentrations of OPE (0, 25, 50 100 μg/mL). 48 h post-incubation, A20 cells were collected and washed once with PBS. A20 cells were incubated with 5 μL Annexin V-FITC and 5 μL PI in 200 μL 1 × binding buffer. Then 200 μL 1 × binding buffer was added to each sample. Samples were analyzed on a flow cytometer (BD Biosciences) and the data was analyzed using the CytExpert software (BD Biosciences).

### Western blot analysis

A20 cells were exposed to different concentrations of OPE (0, 25, 50, and 100 μg/mL) for 48 h. A20 cells were resuspended in RIPA buffer with proteinase inhibitors (Sigma, USA) and incubated on ice for 20 min. The lysate was then centrifuged at 12,000 rpm at 4°C for 20 min. The supernatant was collected and the protein concentration was determined with the BCA protein Assay Kit (Thermo Scientific, USA). Total proteins from different samples were separated and transferred to PVDF membranes (Millipore, USA). The membrane was blocked with 5% milk in TBS-Tween for 1 h at room temperature, and was incubated with primary antibodies at 4°C overnight. After 3 washes with TBS-Tween, the membrane was incubated with HRP-conjugated secondary antibodies at room temperature for 1 h. An enhanced chemiluminescence (ECL) kit (Millipore, USA) was used to detect the protein bands and Image J software was used to determine the relative protein expression.

### Animal experiments

Male Balb/c mice (6–8 weeks, 16–20 g) were housed in-group in cages at a mean constant temperature (25 ± 2°C), humidity (55 ± 5%) and illumination (12 h light-dark cycle), and free access to standard pellet chow and water. The animal experiments were approved by the Institutional Animal Care and Use Committee of Foshan University. 5 × 10^6^ A20 cells in PBS were subcutaneously injected into the right oxter of Balb/c mice. When the tumors reached 50~100 mm^3^, mice were randomly divided into three groups: the control group, low-dose group and high-dose group (*n* = 4). Mice in the control group were gavagely administered with PBS. Mice in the low-dose group were gavagely administrated with 15 mg/kg OPE, while mice in the high-dose group was gavagely administrated with 45 mg/kg OPE every other day. The administration was continued for 10 days. At the end of experiment, rats were euthanized with CO_2_ and tumors were isolated.

### TUNEL analysis

Tumor tissues were fixed and embedded in paraffin. Tissue sections were cut, deparaffinized, repaired with protease K, and permeabilized. Then sections were stained with the Fluorescein (FITC) TUNEL Cell Apoptosis Detection Kit (Servicebio, Wuhan, China). The nuclei were stained with DAPI. Tumor sections were visualized under a fluorescence microscopy (Zeiss, Germany).

### Immunohistochemistry

Tumor tissues were fixed with 4% paraformaldehyde and embedded into paraffin. Paraffin sections (4 μm-thick) were deparaffinized, antigen-retrieved, and treated with 3% hydrogen peroxide. Then, sections were blocked with 3% BSA, followed by incubation with primary antibodies (cleaved casepase-3 and Ki-67 antibodies, Cell signaling technology) overnight at 4°C. After washed with PBS, sections were incubated with HRP-conjugated secondary antibodies and color was detected using a DAB detection kit. Sections were counterstained with hematoxylin. Three random fields per tumor were selected and the number of positively stained cells per 40×field were counted.

### Statistical analysis

All experiments were repeated three times and the data were represent as means ± SD. Comparisons among two groups were analyzed using unpaired two-tailed Student’s *t*-test. Comparisons among more than two groups were performed by the one-way analysis of variance (ANOVA) using the SPSS 19.0 software. A *p* < 0.05 was considered statistically significant.

### Ethics approval and consent to participate

All animal experiments were conducted in compliance with the Chinese legislation in the Guide for the Care and Use of Laboratory Animals. The experiment was approved by the Ethical Review Committee of the Experimental Animal Welfare, Foshan University.

## Supplementary Materials

Supplementary Figures

Supplementary Table 1
