# Supplementary material for: In vitro and in vivo anti-lymphoma effects of Ophiorrhiza pumila extract
Source: Aging (Albany NY). 2022 May 3;14(9):3801–12. doi: 10.18632/aging.204041 (PMC9134945; doi:10.18632/aging.204041)
Supplement: Supplementary Figures [file aging-14-204041-s001.pdf]

SUPPLEMENTARY FIGURES

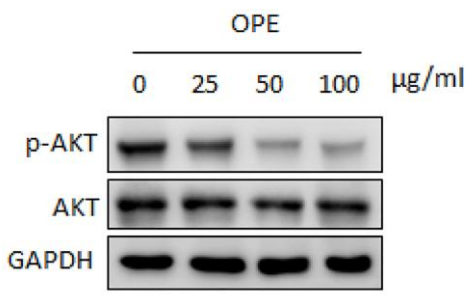

Supplementary Figure 1. Western blot analysis of the expression and phosphorylation of AKT in A20 cells following OPE treatment.

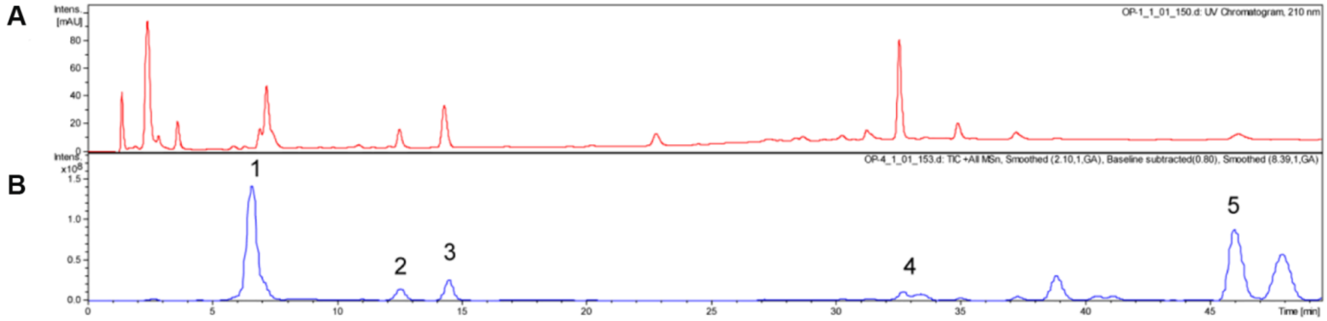

Supplementary Figure 2. HPLC-MS^2 analysis of the OPE. (**A**) UV chromatogram (210 nm). (**B**) Total ion chromatogram (positive ion mode).
