# Supplementary material for: In vitro and in vivo anti-lymphoma effects of Ophiorrhiza pumila extract
Source: Aging (Albany NY). 2022 May 3;14(9):3801–12. doi: 10.18632/aging.204041 (PMC9134945; doi:10.18632/aging.204041)
Supplement: Supplementary Table 1 [file aging-14-204041-s002.pdf]

SUPPLEMENTARY TABLE

Supplementary Table 1. Compounds identified from the chromatogram of OPE by HPLC-MS^2.

Peak	Retention time (min)	MS (*m/z*)	MS^2 (*m/z*)	Tentative compounds	Relative peak area (%)
1	7.2	513.26	351.16	pumiloside	4.37
2	12.6	497.25	335.19	deoxypumiloside	3.39
3	14.5	349.11	337.19	camptothecin	8.48
4	32.5	383.26	327.15	aknadinine	16.0
5	46.1	413.31	301.11	*β*-stigmasterol	2.42
